# Dr. Frank C. Clarke

**Published:** 1916-09

**Authors:** 


**Dr. Frank C. Clarke** of Catskill, N. Y., Buffalo 1873, died at Palenville, May 30, aged 64.

